# Mississippi Valley Dental Association

**Published:** 1884-04

**Authors:** F. W. Sage


### MISSISSIPPI VALLEY DENTAL ASSOCIATION.

### FORTIETH ANNUAL MEETING, HELD AT CINCINNATI, MARCH 5TH AND 6TH, 1884.

PHONOGRAPHICALLY REPORTED FOR THE INDEPENDENT PRACTITIONER.

BY F. W. SAGE, D. D. S.

*Wednesday Morning, March 5th.*

After organizing, a paper, illustrated by blackboard drawings, was read by Dr. G. W. Smith ; Subject, " Treating Nerve Canals of Dead Teeth." This was supplemented by remarks by the author on mounting porcelain crowns, in which he dwelt on the beauty and utility of gold bands and backings as a support for the crown, and urged the importance of devoting ample time to each case, in order to secure a perfect result. Dr. W. S. How followed with an illustrated description of his method of mounting crowns. Dr. H. A. Smith remarked, later, that in certain cases where the roots were broken or decayed considerably below the gum line and presented a bell-shaped opening, he had found difficulty in securing a proper alignment of the platinum screw-post with the porcelain crown. In these cases he departed from the detailed instructions of Dr. How, and overcame the difficulty by attaching the post with cement to the crown, and then forcing both to their final position at once, then removing the cement and proceeding as per instructions. He liked this method of Dr How, because he had found by experience that it enabled the operator to proceed by clearly-defined steps to a final result, satisfactory to himself and his patient. It is of vital importance, he has found, that the concavity on the end of the prepared root be made rather deep, so as to prevent any rotation of the artificial crown. The time reqnired for the operation, after having the root prepared, is from one to two hours.

*Dr. F. A. Hunter*—For many years I have made a general practice of mounting teeth on roots, employing various methods from time to time. I consider Dr. How's method the most feasible, and the best that has hitherto been brought to my notice. I have mounted perhaps ten or a dozen of his crowns within a year, and I consider the system very complete, because you have things under such perfect control. In the case of bicuspids, a porcelain face can be put on stronger and better than by any other method, to my knowledge and in my judgment. It has proven very satisfactory in my hands. Dr. Smith has directed attention to the great importance of hollowing out the root for the reception of the porcelain crown, to a sufficient depth to insure the latter's not turning on its axis. [This refers to the concavity conforming to the festoon of the gum.—Rep.] That is of indispensable importance to the stability of the crown, and should not be overlooked.

*Dr. J. Taft*—I have never tried Dr. How's method of mounting crowns. It is similar to many other methods in that if all the steps are well taken, good results may be obtained. It has perhaps more advantages than some others. One of these is the facility of adapting the crown and then securing alignments. Many operators are in too great haste to accomplish the best results. Take such a case as the one in the diagram before you. [A lateral root, decayed a line or more below the border of the gum.—Rep.] We find, in attempting such an operation, that there are difficulties we did not suspect. Dr. How has, no doubt, a great many little turns and resources which would not at once occur to a novice. Although the operations might be performed under his very eyes, by Dr. How, many little details would probably escape his notice. He would need to improvise and work out other details, in addition to all he had learned from close observation, before he would be possessed of that peculiar facility which would enable him to proceed uninterruptedly to the achievement of a successful result. In regard to instances of very badly decayed roots, perhaps much is attempted which would better not be attempted at all. Where there is a marked predisposition to inflammation, I should hesitate to make any more than a merely experimental operation. Take a person whose health is good, who is well nourished, and you can do almost anything in the way of operations which in the case of another would be wholly unadvisable. You could almost put a pivot into the socket of an extracted root in one case; in another, you might well hesitate to even probe the pulp canal. We should select our cases in accordance with the indications of diathesis. Where there is manifestly diseased condition of the gum, in many cases, the contact of a filling material above the gum line, as in the case in the diagram, would not be tolerated. At all events, where amalgam or any other plastic is used about the ends of the roots above the gingival border, great care should be taken to finish it smoothly and flush with the margin of the root. It is important to have the gum in a healthy condition; where there is tumefaction or hypertrophy, the operation should not be attempted at all.

*Dr. H. A. Smith*—I approve of all that Dr. Taft has said. In what I said about the difficulty of attaching the crown to those badly decayed roots, I had particularly in view the mechanical aspect of the case, and expressed no opinion as to the advisability of attempting the operation, regarding the case from a pathological standpoint.

*Dr. How*—Of course there are limits to all apparent possibilities. In actual practice, no doubt, efforts are often misdirected to the salvation of roots which ought to be extracted.

*Dr. G. W. Smith*—Dr. How's method of mounting crowns is certainly deserving of high praise, even granting that it does not render all the hitherto impossible cases possible.

*Dr. Sillito*—I have set a number of crowns by Dr. How's method, and am pleased with the results I have been enabled to attain. In one instance, where I failed in my endeavors to mount a crown by other well-known means, by resorting to this method I succeeded in fixing on a crown which has now done good service for eight months.

*Dr. Jennings* (*sotto voce*)—It will be delivered next month. (Laughter.)

*Dr. J. Taft*—Dr. How recommends pressing back the gum with cotton and sandarac varnish, as is often done in other operations, in order to fairly expose the end of the root. I presume that he continues the process of expansion until he has secured sufficient space to admit of finishing the plastic filling above the gum line. Now that needs to be done carefully, so as not to excite inflammation. By prolonged pressure you are liable to induce a condition not easy to control. It is commonly expected that only those who have full knowledge of all these things should do the talking. I should like to hear from some who do *not* know.

*Dr. C. M. Wright, Chairman*—Shall the chair call out some who do not know?

*Dr. Taft*—Yes, if you please.

*Dr. Wright*—Dr. Cassidy, will you please expose your ignorance? (Laughter.)

*Dr. Cassidy*—I know *too* much. (Renewed laughter).

*Dr. J. Taft*—Dr. Corydon Palmer carved out and attached a crown of walrus tusk to supply the loss of a molar crown in his own mouth. It was attached with screws so skillfully as almost to defy detection. That was many years ago.

*Dr. Berry*—I have always been afraid of trouble when setting crowns on diseased roots, and yet I have frequently done it. I always tell the patient beforehand that he may have trouble. When I undertake such operations I want some assurance of my patient's confidence in my integrity, so that in case of failure I will not be unreasonably blamed.

Calls for Dr. Jennings, who failed to respond.

*Dr. Frank Hunter*—I consider it a marked breach of etiquette, not to say an outrage, that a man should come down here from Northern Ohio and sit in this assembly, absorb all that is said, and never open his mouth. Can't somebody make him get up? (Laughter.)

*Dr. Jennings*—I am paying my way and I want my money's worth, first. (Laughter.)

*Dr. Sage*—I have not yet tried Dr. How's crowns, although I have several times thought I would do so. Have had a difficulty (of which I have heard others complain) in using amalgam about the Bonwill pins in roots, in that the mercury amalgamates with the pin, and the latter soon breaks. The method described in Richardson's Mechanical Dentistry, credited to Dr. Edwin T. Darby, is, though nothing new, an excellent one (when properly performed) for all front teeth. It consists of a strong platinum wire, fitted into the root, over this a thin cap of platinum, covering the end of the root accurately, a plain plate tooth backed with platinum, and finally soldered to the pin and the platinum cap with pure foil scraps. The appliance admits of proper alignment, is more cleanly than a wooden-pivoted crown, much more accurate in adaptation, and much stronger. A large wire should be used, or there will be danger of its bending. A smaller wire of stiffened gold may be used, but more care will be required in soldering. The various steps of the operation are easily understood and mastered, and with ordinary care no uncertainty attends the final result. When you have finished the appliance and place it in the mouth, you find that it fits exactly. It may be reset, usually in a few moments. The peculiar advantage of this method is that if there be any failure it is usually simply a failure of attachment of the pin in the root, which is easily remedied. The appliance is usually found unimpaired, and all that is required is ten or fifteen minutes for resetting. This seldom occurs before the lapse of from three to five years. I use a rather large platinum post (made to order) in the root, and it never bends. At present, the advantage of having a wide range for selection from either plain, plate or rubber teeth, gives this method the preference over some others. The method is not quite perfection, but what other method of mounting crowns is free from all objection?

*Dr. Van Antwerp*—Spoke of the importance of a deep concavity in preparing a root for a How's crown.

The subject under consideration being passed, the Chairman, Dr. Wright, read a paper, entitled "Phosphate of Lime and the Teeth."

*Dr. A. O. Rawls*—The administration of complementary food is not generally well understood. The elements contained in natural food are supplied directly to the organs and tissues, in a degree more or less discernible. It is not sufficient that the food appropriate for the nourishment of tissues be merely supplied; to accomplish the desired object assimilation of such food is requisite. We know that complementary food administered to a pregnant woman will, under favorable circumstances, benefit her and the child as well, but how many women will you find who are willing to submit to the conditions favorable for the accomplishment of the results desired? Exercise is the great requisite, and indeed it would seem to be all-sufficient to insure health in the child; certainly without it the mere administration of complementary food will hardly avail to improve matters. Tonics administered to increase the neural circulation, or the blood circulation, will accomplish nothing without exercise. Among the children of the better classes, those who are carefully kept in-doors and at school, you will find not so much evidence of a deficiency of lime-salts in the blood, but the frail character of their teeth must be attributed to a lack of free out-of-door exercise; in short, to defective nutrition. They perhaps subsist on delicate viands, pulpy food, and their teeth suffer for lack of the exercise which nature designed them to have. Use one side of the mouth exclusively in masticating, and in time the opposite teeth will suffer from the neglect of exercise. The complementary foods in the shape of medicines have their place, but I apprehend that they are not unfrequently prescribed for patients, when a prescription calling for plenty of light, air and exercise would be more suitable.

*Dr. H. A. Smith*—There are many crude experiments made in the use of phosphate. Man is peculiarly constituted, physically, in that he is omniverous. Certain races subsist on meat entirely, others on fish, others again on vegetables and fruits. Which of all these classes has the best teeth? Is it the Icelander or the Greenlander, whose diet is almost exclusively meat; or is it the macaroni-eating Italian, or the rice-eating Chinaman, or the English or American, whose diet is various? I was interested, some time since, in certain observations and comparisons made by an eminent English agriculturist and scientist. He states that the English wheat-grain, which is larger and plumper than the American wheat-kernel, contains a larger per cent of albuminoids. The tabular statement is to the effect that the English grain contains 13 per cent of albuminoid, or starchy substance, the American grain, 11.95 per cent, and the wheat of the Pacific Coast, 8 or 10 per cent only. Now it is the starchy food which he claims is the most valuable. We on this side of the water have generally held the belief that the outer shell which contains the gluten or albumen is the most nutritious part of the grain.—Dr. Smith showed the hexagonal structure of the internal portion of the grain, also the arrangement of the several layers composing the shell, by illustrations. There are six layers. These contain the phosphates. Taking into consideration the contrasts which are discovered by an examination of the nature of foods upon which the races appear to thrive the world over, the question, what food is best adapted to promote the development and strength of osseous structures, would seem to be involved in much that is obscure and perplexing. He surmises that in the instance of a pregnant woman, the nutritive process by which the fœtal structure is developed is analogous to the development of the chick in the shell. The inner layers of the shell are appropriated by the developing embryo, so that the shell, becoming more and more thinned, admits finally of the release of the inmate at the proper time. So in all probability the child draws on the mother for bone-making material. Dr. Smith cited the case of his own children. Three, who always ate bread made from bolted flour, have good teeth, while the teeth of the fourth, who early exhibited a decided preference for brown bread, are the poorest of all. It is worthy of note that while the urine of the adult often contains phosphates, that of the child contains none.

*Dr. G. W. Smith*—What do you think of milk?

*Dr. H. A. Smith*—Give the child all the milk it wants. Milk contains a larger amount of the phosphates than meat or the cereals.

*Dr. G. W. Smith*—Knew a child seven years old who had "never eaten anything but milk," and whose teeth were excellent.

*Dr. Van Antwerp*—Dr. Johnson, the author of the dictionary, remarked that in England oats were used to feed horses, and in Scotland to feed men. Some one replied that that accounts for the excellence of the horses in England and of the men in Scotland. The Scotch, in the Highlands, have good teeth. In Glasgow they have bad teeth—no better than the Londoners. Air and exercise no doubt have much to do with this.

*Dr. H. A. Smith*—An observer states that he saw many bandy-legged children in the region of Glasgow. A similar fact was noticed about Iceland. The lime-salts in the water used are probably scanty.

*Dr. C. M. Wright, Chairman*—Dr. Cushing, of Chicago, made the remark that he believes from his clinical observations that the exhibition of the lime-salts would sometimes assist a woman in pregnancy when the natural food would not assimilate.

*Dr. Sage*—It has been shown that at the very time the fœtus was supposed to be appropriating more than its share of the lime-salts from the mother, a free elimination of the salts from the system was going on. In this condition of the mother's system would the exhibition of artificially prepared lime-salts be indicated? Much diversity of opinion as to the efficacy of this artificial means for repairing the waste, prevails among scientists in the medical faculty.

*Dr. Osmond*—It was formerly, and is still the practice with many, to administer the hypophosphites in uninterrupted doses to pregnant woman. That is a mistake. I administer the medicine for two weeks, and then allow a lapse of two weeks. At the end of the fortnight some nausea may be noticed. I have again and again seen the hypophosphites administered to pregnant woman whose teeth were poor, with the result of securing for the child a good set of teeth in after years. As to the effects of lime-water, at a certain watering place I visited in England, in 1880, the teeth of those living in the vicinity were very poor. The bicarbonate of lime in the springs readily induced petrifaction upon articles placed in them, and yet the teeth of the dwellers there soften rapidly. What are we to infer from that?

The principal constitutent of our teeth is the phosphate of lime. Generally speaking, it is certain that in some regions where the water is very hard, teeth are very bad. In other regions, where the hardness of the water is not produced by bicarbonate of lime, there is plaster-of-paris formation, which is less noxious. The sulphate of lime has a different effect upon the teeth from that produced by the carbonate of lime.

THURSDAY MORNING SESSION.

The subject announced for discussion was: "What means are best adapted for maintaining the teeth and mouth in a healthy condition?"

*Dr. Taft*—Opened the discussion of the subject by calling attention to the duty of the dentist to instruct the patient about the care of his teeth. The dentist should not shrink from the performance of disagreeable work. In all professions there is the scavenger's work to be done.

*Dr. Osmond*—Has discovered that many who use the brush use it in the morning only. He always begins his course of treatment by attention to the removal of tartar, etc., and often waits a day or two before further operations. Recommends the use of tooth-powder at night particularly. Water alone may be used in the morning.

*Dr. H. A. Smith*—Recommends the use of Listerine as a mouth-wash. He spoke of the early discovery of bacteria in the mouth. On September 14th, 1683, Antony Van Lieuwenhoek, of the Netherlands, gave notice to the Royal Society of Great Britain that he had seen with the microscope little animals moving in a lively manner in the white substance scraped from his teeth—the first bacteria ever seen with the human eye. He described several varieties, easily recognized as *Bacillæ*, *Bacterium*, *Leptothrix Buccalis*, etc. He wonders, since he cleans his teeth, how so many creatures manage to live in his mouth! He found after drinking hot coffee that they had disappeared. After awhile they reappeared. In 1692 he made accurate drawings of these various animalculæ (fungi), which to this day have not been surpassed. He (the speaker) doubts whether the presence of these parasites in the mouth is of important significance.

*Dr. Berry*—A celebrated microscopist told me he had discovered bacteria in a drop of his own saliva. His mouth was in a very unhealthy condition. Conjoined suppuration of the alveolus and the gums, as the old writers called it, was noticeable. He lost several of his teeth finally.

*Dr. G. W. Smith*—Bacteria are not peculiar to a diseased condition of the mouth; they are found in perfectly healthy mouths. Whenever accumulations are found about the teeth bacteria may be discovered in abundance. They are not always destructive. But if the mouth is neglected, they may be very destructive.

*Dr. Jay*—I have more than once sent patients home to brush their teeth and clean their mouths before I would operate for them. Too often the dentist's reputation is made to suffer when the real blame lies with the negligent patient. Too many of our patients fail to attend to our injunctions.

*Dr. Osmond*—By way of a gentle hint I have sometimes suggested to a patient the advisability of getting weighed before having tartar removed. After having had the tartar removed and the gums treated, I recommend the use of a rather soft brush. To avoid injury to the tender gums I advise a brush with the outer rows of bristles cut away. An unbleached Russia bristle brush is best. It will outlast half-a-dozen bleached bristle brushes. The bristles of these brushes are wired in, and are consequently more durable. I obtained a lot of these unbleached bristle brushes from a London firm, but there is now an American firm manufacturing the same brush.

*Dr. Taft*—Of all the antiseptic mouth-washes there is perhaps nothing better than Listerine. I have used it for about a year.

*Dr. Jay*—How do you use it?

*Dr. Taft*—Like other washes, upon the brush. It is especially useful as a wash, to be used before retiring. Those who sleep with the mouth closed have, perhaps, less need of a brush than many others. At all events their teeth will decay less rapidly. At night the watery portions of the saliva are thrown out, and the mucus becomes agglutinated, and injury to the teeth results. The presence of bacteria in the oral cavity is, perhaps, of little importance; still, as an essential of cleanliness their removal is desirable, and this may be accomplished by using Listerine. I do not, however, regard them as a prime factor in decay.

*Dr Osmond*—I do not regard bacteria as worthy of any very particular consideration. Parasites abound in all parts of the system.

Large fleas had little fleas,
And those have fleas to bite 'em,
And there are fleas to feed on these,
And so, *ad infinitum.*

(TO BE CONTINUED.)

